# Lipoid Pneumonia Secondary to Diesel Aspiration: An Occupational Hazard

**DOI:** 10.7759/cureus.58509

**Published:** 2024-04-18

**Authors:** Gaurang M Aurangabadkar, Sumer S Choudhary, Shafee M Khan

**Affiliations:** 1 Respiratory Medicine, Datta Meghe Medical College, Datta Meghe Institute of Higher Education and Research (Deemed University), Nagpur, IND

**Keywords:** bronchoalveolar lavage, computed tomography, bronchoscopy, chemical pneumonitis, lipoid pneumonia

## Abstract

Lipoid pneumonia is defined as a type of pneumonia that occurs as a result of inhalation of either endogenous or exogenous lipid-containing products in the lungs. We present the case of a 55-year-old male patient who presented with chief complaints of cough with blood-tinged sputum, right-sided chest pain, dyspnea, and fever for two days. The patient gave a history of working as a mechanic in an automobile garage and reported an episode of accidental aspiration of diesel during diesel siphoning at the workplace. A chest X-ray and computed tomography (CT) scan of the chest were done, which revealed right-sided lower lobe consolidation. The patient was admitted and started on intravenous antibiotics, corticosteroids, and inhaled bronchodilators, along with oxygen support. A bronchoscopy was done, which revealed the presence of thick mucoid secretions in the right lower lobe bronchus. The patient was discharged after 10 days with stable vitals and was advised to have regular follow-ups to monitor for any long-term pulmonary complications.

## Introduction

Lipoid pneumonia is typically described as a type of pneumonia that occurs as a result of the accidental inhalation or aspiration of lipid-containing material into the lungs [1]. This terminology of lipoid pneumonia was first introduced by Laughlen in 1925, after conduction of multiple autopsies on patients who had a chronic history of using oil-based medications [2]. Lipoid pneumonia arises as a result of blockage of the distal airways and the alveoli with lipid-containing products that lead to an inflammatory reaction in the airways, further interfering with normal gas exchange in the alveoli. This is a rare form of pneumonia, with characteristic histopathological and radiological findings, with the main clue being a personal history of long-term use of oil-containing medications or an occupational exposure to diesel, petrol, or other hydrocarbon fuels in patients working in the automobile, petroleum, and automotive repair industries. Primary care physicians as well as specialists should be aware of this condition due to the high probability of the patient developing life-threatening and often fatal hypoxemic respiratory failure.

## Case presentation

We present the case of a 55-year-old male patient who presented to the respiratory physician with chief complaints of severe dyspnea, cough with mucoid expectoration with occasional blood-tinged sputum, fever, and right sided pleuritic chest pain, which was more on deep inspiration. A detailed history revealed that the patient worked as a mechanic in an automobile garage, and had a history of accidental aspiration of diesel during the process of diesel siphoning, two days ago, after which he started to develop significant respiratory complaints as described above. A clinical examination was done, which revealed the following findings (Table 1).

**Table 1:** Summary of the clinical findings of the patient

Clinical parameter	Clinical findings in the patient
Temperature	102.3 degrees Fahrenheit (F)
Pulse rate	116 beats per minute
Respiratory rate	30 breaths per min
Blood pressure	130/70 millimeters of mercury (mm Hg)
Oxygen saturation	90% on room air
Chest auscultation	Crepitations heard in the right infra-axillary and infrascapular regions of the chest wall

**Figure 1:**
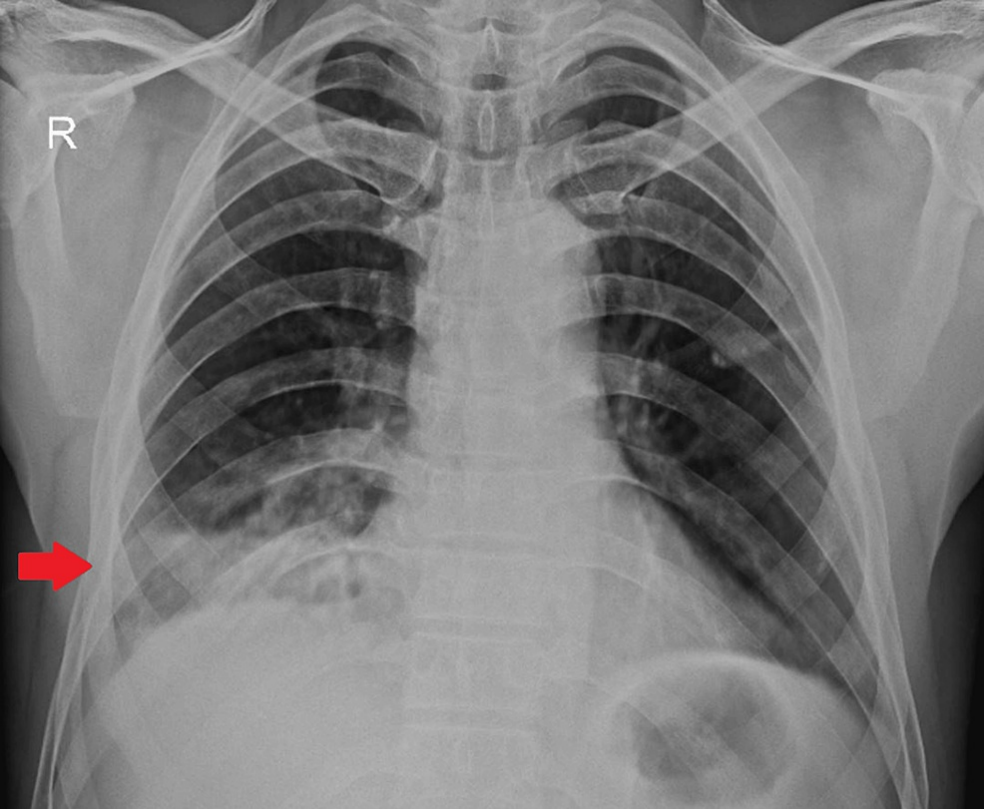
Chest X-ray posteroanterior view showing haziness in the right lower zone (red arrow)

A chest X-ray posteroanterior (PA) view of the patient was done, which revealed the presence of haziness in the right lower zone (Figure 1).

**Figure 2:**
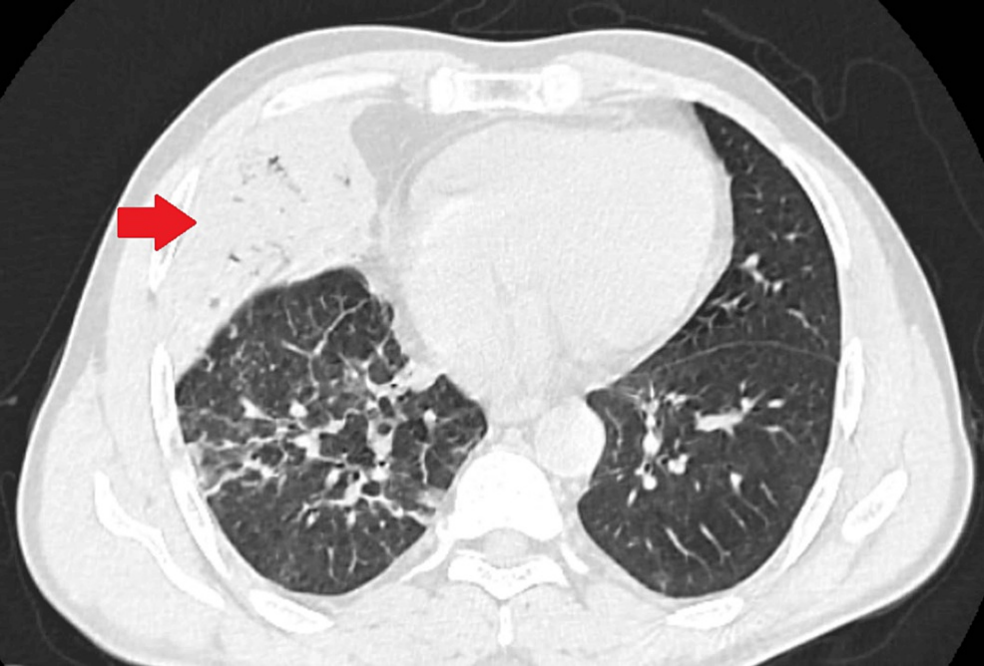
Computerized tomography scan of the chest; axial view, showing the presence of airspace consolidation in the right middle lobe with air bronchogram suggestive of right-sided pneumonia (red arrow)

For further evaluation, a CT scan of the chest was done which illuminated the presence of an airspace consolidation in the right middle lobe with subsegmental atelectatic changes in the right lower lobe; axial view (Figure 2).

**Figure 3:**
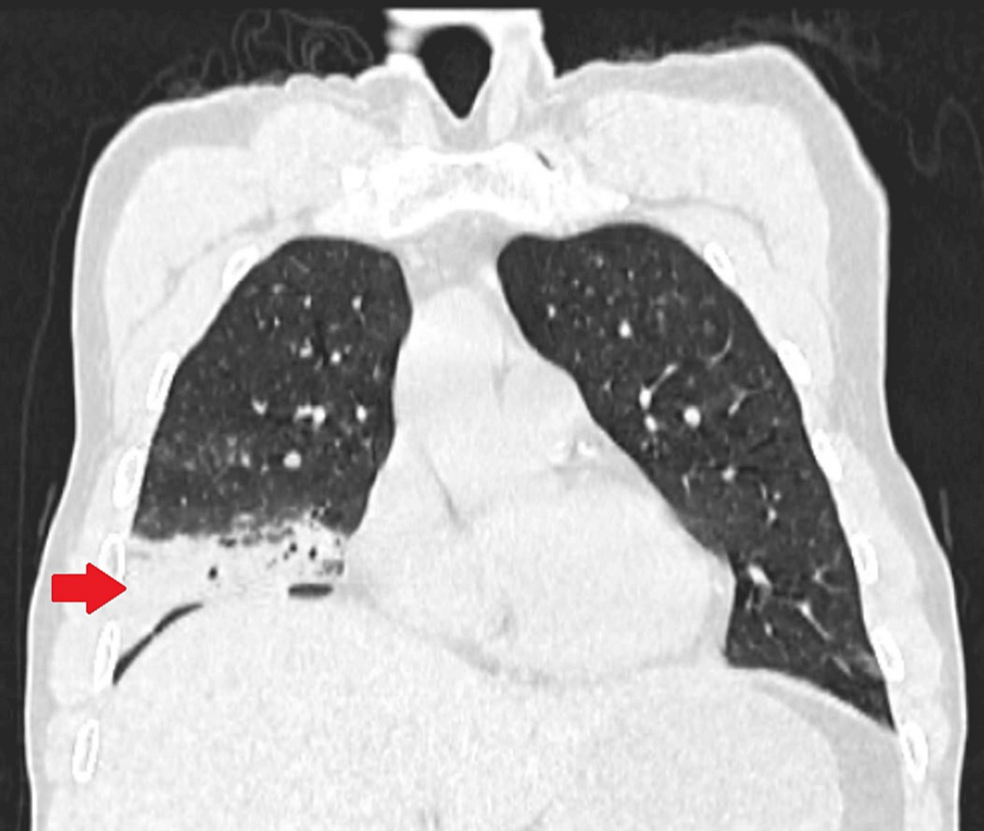
Computerized tomography scan of the chest; coronal view, showing the presence of consolidation in the right middle and lower lobe (red arrow)

The CT scan of the chest shows right middle and lower lobe consolidation; coronal view (Figure 3). 

The patient was admitted to the intensive care unit (ICU) and started on oxygen support, intravenous broad-spectrum antibiotics, corticosteroids, and nebulized bronchodilators. A flexible fiber-optic bronchoscopy of the patient was done which revealed the presence of thick mucoid secretions in the right middle and lower lobe bronchus segments. Bronchoalveolar lavage (BAL) was taken from the right lower and middle lobe bronchus segments and sent for cytology, bacterial culture, and sensitivity. The BAL findings are summarized as follows (Table 2).

**Table 2:** Summary of findings of the various bronchoalveolar lavage fluid investigations of the patient

Lab parameter	Lab findings
Bronchoalveolar lavage: cytology	Presence of foamy macrophages noted
Bronchoalveolar lavage: culture and sensitivity	No pathogenic organisms grown
Bronchoalveolar lavage: Ziehl-Neelsen staining for acid-fast bacilli (AFB)	Negative for AFB

The patient showed improvement in the clinical parameters as well as his symptoms after 10 days of hospitalization, and he was discharged with advice to monitor his oxygen saturation levels regularly, along with regular follow-up after 15 days for further evaluation and monitoring.

## Discussion

Lipoid pneumonia is described as a rare etiological factor leading to the development of pneumonia, which was initially detected in geriatric patients with a history of the use of oil-based laxatives as well as in patients of the pediatric age group who used nasal drops that contained mineral oil [3]. The other risk factors can include neuromuscular disorders, deranged anatomy of the nasopharynx, which lead to an increased predisposition towards aspiration, and a higher occupational risk amongst “fire-breathers” who perform stunts in circuses or entertainment shows [4], as well as the siphoning of hydrocarbon fuels such as petrol and diesel from vehicles, which is a common practice amongst mechanics working in the automotive industry in developing countries.

The pathophysiological mechanisms involved in the development of lipoid pneumonia consist of a chronic inflammatory reaction to the presence of lipids in the airways and the lung parenchyma. These lipid-containing products reach the airways after bypassing the normal cough reflex, secondary to accidental aspiration or inhalation. The fat-containing substance is engulfed by the macrophages, leading to the formation of foamy macrophages. However, a vicious cycle ensues in the airways as the macrophages lack the ability to metabolize the fat, leading to a granulomatous reaction secondary to repeated exposure of the airways to the lipid-rich material [5].

Clinically, a patient with lipoid pneumonia can present with both acute and chronic symptoms, often directly proportional to the magnitude of exposure to the offending agents. A patient with low-dose exposure to lipids can present with a history of chronic productive cough, fever, weight loss, and chest pain, which often mimics clinical presentations seen in patients with chronic infections such as tuberculosis (TB). On the other hand, high-dose exposure to the offending agent can lead to the development of severe hypoxemic respiratory failure, which may progress towards acute respiratory distress syndrome (ARDS) [5]. The diesel siphoning is usually done with the back leaning in a forward direction, which predisposes the middle lobe towards being the commonest site affected in lipoid pneumonia [6], which was identical to the findings seen in our case. The commonest findings on a CT scan of the chest in adult patients with exogenous lipoid pneumonia were found to be consolidation of the airspaces (predominantly in the middle and lower lobes), nodular parenchymal lesions, as well as the “crazy-pavement” appearance [7].

The mainstay of treatment for lipoid pneumonia remains avoidance of the offending agent, adequate oxygen support with antimicrobial coverage for secondary bacterial infection, administration of corticosteroids to inhibit the inflammatory cascade in the lungs, and other supportive measures such as chest physiotherapy to facilitate drainage of the tracheobronchial secretions [8].

## Conclusions

Lipoid pneumonia is a rare variant of pneumonia, which is most commonly seen with occupational accidental exposures to hydrocarbon fuels, amongst other causes. A patient who has acute exposure to a high concentration of the offending agent can develop severe respiratory distress with ARDS. Adequate awareness of this rare condition is essential for primary care physicians as well as occupational health experts, to ensure that adequate and early management is instituted for such patients to prevent the progression to ARDS, and eventual fatal outcomes. It is our sincere wish that such dangerous practices of siphoning hydrocarbon fuels by the employees working in the automotive industry in the developing countries should completely stop and that stringent regulations are formulated by the industrial safety authorities of the respective governments of the developing countries, to ensure the safety and well-being of industrial workers.
